# Prevalence and risk factors of caregiver reported Severe Early Childhood Caries in Manitoba First Nations children: results from the RHS Phase 2 (2008–2010)

**DOI:** 10.3402/ijch.v72i0.21167

**Published:** 2013-08-05

**Authors:** Robert J. Schroth, Shelley Halchuk, Leona Star

**Affiliations:** 1 Faculty of Dentistry, Department of Preventive Dental Science, University of Manitoba, Winnipeg, Canada; 2 Faculty of Medicine, Department of Pediatrics and Child Health, University of Manitoba, Winnipeg, Canada; 3 The Manitoba Institute of Child Health, Winnipeg, Canada; 4 Assembly of Manitoba Chiefs, Winnipeg, Canada

**Keywords:** early childhood caries, dental caries, First Nations, Indigenous, child, preschool, infant and toddler

## Abstract

**Objectives:**

The high prevalence and severity of caries among Canadian First Nations children is a growing concern. Dental surgery in hospital is often necessary to treat the signs of decay but does not address the underlying factors contributing to its development. The purpose of this study was to determine the prevalence and risk factors of caregiver-reported Baby Bottle Tooth Decay (BBTD), or Severe Early Childhood Caries (S-ECC), among preschool children recruited in Phase 2 of the First Nations Regional Longitudinal Health Survey (RHS).

**Study Design:**

Cross-sectional study including interviews with caregivers.

**Methods:**

This study was limited to data from Manitoba First Nations participating in the RHS Phase 2 (2008–10). Data were restricted to caregiver interviews for their child <72 months of age. The main variable of interest was caregiver-reported BBTD, an antecedent term for S-ECC. Data analysis included descriptive statistics and bivariate analyses; p≤0.05 was significant.

**Results:**

Overall, caregivers of 431 preschool children responded. According to caregiver reports, 102/410 (24.9%) children had S-ECC. Further, 65.0% responded that their child had already undergone treatment for caries. Children with S-ECC were significantly older than those without. S-ECC was also associated with paternal education levels and employment status, and maternal smoking during pregnancy. Breastfed children were less likely to have S-ECC, while consuming drink crystal beverages in bottles, and daily intake of soft drinks, juice, sweets and fast food were associated with increased risk. Those who reported that healthcare services were not available and were not culturally appropriate were significantly more likely to have children with S-ECC.

**Conclusions:**

Caregiver reports suggest that nearly 1 in every 4 children has been affected by S-ECC. Identified risk factors for Manitoba First Nations children included age, education and employment, dietary practices, access to care, and disruption to family and culture. This local evidence can be used to help inform future caries prevention activities in these Manitoba communities.

Early Childhood Caries (ECC) is defined as any caries experience in the primary dentition in those <72 months of age (1). Indigenous children around the globe are disproportionately affected by ECC and a more aggressive form of the disease, termed Severe Early Childhood Caries (S-ECC). The prevalence of caries is high among American Indian and Alaska Natives (AIAN), Australian Aboriginals and the Maori (2–6). This reality also exists in Canada where the prevalence of caries in Aboriginal children is staggering (7–12). In Canada, the term Aboriginal encompasses First Nations, the Métis and Inuit.

The problem of S-ECC among First Nations children is an ongoing public health concern (10). In some northern communities, as many as 90% of preschool children may be affected (7,13). For many, dental surgery in hospital is the only option to treat the signs of disease. Unfortunately, this approach does not address underlying factors that contribute to its development. The rates of dental surgery among First Nations children are high for a theoretically preventable problem (14,15).

Several antecedent terms have been used to describe S-ECC, many attributing causation to inappropriate infant-feeding methods (16). One of the most recognized terms is Baby Bottle Tooth Decay (BBTD) (16). The term conjures up images of severe decay affecting the primary maxillary incisors as a result of inappropriate bottle-feeding. In reality, like other chronic diseases, ECC and S-ECC are multifactorial. These new terms were adopted in the late 1990s to refocus attention on the broader factors that influence oral health (17).

While the etiological triad for caries includes teeth, microorganisms and fermentable carbohydrates, there are numerous oral environment factors and personal and lifestyle behaviors at play (18). Some factors that appear to be determinants of S-ECC in First Nations children include limited access to care, the absence of fluoridated water, finances and parental education levels, and oral health literacy (4). In addition, the value that parents place on primary teeth and views on prolonged bottle-feeding have also been identified as predictors of caries-risk (16). Another interesting prevention paradox used to be the recommendation by Health Canada to avoid fluoride toothpaste use until children were 3 years of age. This has likely contributed to the problem of S-ECC in First Nations communities. Daily toothpaste use is now recommended for children in areas where water fluoridation is lacking, like on-reserve communities (19).

S-ECC should be a concern as it increases the risk for caries throughout childhood (20,21) and can impact overall health and well-being (4,22–25). Identifying specific risk factors in First Nations children is useful to help inform targeted prevention and risk minimization activities in the community. This is critical in order to reduce the incidence and severity of caries in future generations. The purpose of this study was to determine the prevalence and risk factors of caregiver-reported BBTD (i.e. S-ECC) among Manitoba children participating in Phase 2 of the Regional Longitudinal Health Survey (RHS).

## Methods

The RHS is a national survey of First Nations persons in Canada. Phase 1 was conducted in 2002–2003 while Phase 2 occurred during 2008–2010 (26,27). It is the only national survey of its kind and was designed, developed and delivered by First Nations people. RHS was intended to serve as a holistic survey on physical, mental, spiritual and emotional well-being. Oral health was 1 aspect of physical health that was assessed.

Data originated from the RHS Phase 2, which was collected from participating Manitoba First Nations persons between 2008 and 2010. RHS was regionally implemented within Manitoba by the Assembly of Manitoba Chiefs (AMC) and with the approval and guidance of the AMC Health Information Governance Committee (http://amc.manitobachiefs.com/images/pdf/hirgc.pdf). While AMC managed and oversaw the process, communities collected their own data. Community members hired and trained in data and research skills performed data collection. The First Nations Information Governance Centre supported regions in implementing RHS for a national roll-up of data.

The child survey component served as the basis for this study. Data were obtained from structured interviews with parents or caregivers of children. Participants provided written informed consent. As BBTD and S-ECC affect infant and preschool children, we restricted the analyses to children <72 months of age in keeping with recognized case definitions (1,28,29). Caregiver-reported BBTD was the outcome variable of interest. BBTD is an antecedent term no longer in vogue to describe multiple caries lesions in young children now referred to as S-ECC. This term was used in both Phase 1 and Phase 2 surveys as it was believed that this would be a familiar term among participants. However, for our purposes, they are essentially the same. From this point on in this manuscript, we will refer to it as S-ECC to avoid confusion. By definition, S-ECC exists when a child under 3 years of age has caries involving smooth surfaces and when caries affects the primary maxillary anterior teeth in those 3–5 years of age (1,29).

Other variables of interest included child and caregiver characteristics, such as age, sex, caregiver educational and employment status, childhood health status, infant-feeding practices and dietary habits. Other themes assessed included barriers to overall health care access and family histories with residential schools.

Data were analyzed at the AMC Research Centre using PASW Statistics 18 (Chicago, Il). Analysis included descriptive statistics [frequencies and means±Standard Deviations (SD)], *t*-tests and Chi-square analysis. Unadjusted odds ratios (OR) for S-ECC were also calculated. A p≤0.05 was significant. Data were suppressed and not reported when cell sizes were ≤5.

## Results

A total of 431 caregivers of children <72 months of age participated. There was an almost even representation of boys and girls; 51.3% (n=221) were male. The average age of participating children (<6 years of age) was 2.7±1.7 years. Meanwhile, the average age of caregivers was 27.7±10.8 years and the majority were female (86.3%).

While 57.2% indicated that their child had already visited the dentist, less than half of the children (47.4%) were reported to have seen a dental professional within the last year. Only 47.3% stated that their child had no current dental needs. Nearly 1 in every 4 children (24.9%) was reported to have S-ECC and 65% of these children actually underwent some dental treatment. Not surprisingly, children with S-ECC were significantly more likely to need dental extractions (p=0.002). Fortunately, children with S-ECC were significantly more likely to have received dental care within the last 12 months (p<0.001).

### Child and caregiver characteristics and S-ECC

Table I reports some child and caregiver characteristics in relation to S-ECC. Children with S-ECC were significantly older than children who did not to have the condition (3.3±1.4 years vs. 2.5±1.7, p<0.001). There was no significant relationship between S-ECC and the sex of the child (p=0.059). The unadjusted OR suggests that boys were 1.5 times more likely to have S-ECC than girls. The age and sex of caregivers were not associated with the presence of S-ECC (Table I).

**Table I:** Characteristics of children and their responding caregiver in relation to the presence of S-ECC

Variable	Number with S-ECC (%)	Number without S-ECC (%)	p	Unadjusted odds ratio (OR)
Age of child			**0.004**	0.50
≤2 years	36 (18.4)	160 (81.6)		
3–5 years	66 (30.8)	148 (69.2)		
Sex of child			0.059	1.54
Male	60 (28.8)	148 (71.2)		
Female	42 (20.8)	160 (79.2)		
Sex of caregiver			0.98	0.99
Male	13 (24.5)	40 (75.5)		
Female	88 (24.7)	268 (75.3)		
Mother's highest level of education			0.064	1.57
<High school	70 (28.2)	178 (71.8)		
≥High school	31 (20.0)	124 (80.0)		
Father's highest level of education			**0.048**	1.81
<High school	69 (28.3)	175 (71.7)		
≥High school	17 (17.9)	78 (82.1)		
Mother works for pay			0.53	1.18
No	70 (26.1)	198 (73.9)		
Yes	27 (23.1)	90 (76.9)		
Father works for pay			**0.050**	1.68
No	59 (29.9)	138 (70.1)		
Yes	27 (20.3)	106 (79.7)		

Chi-square analysis.

While it appeared that mothers who did not complete high school were more likely to have a child with S-ECC than those who had completed high school, the association was not significant (Table I). Children whose fathers did not complete high school were significantly more likely to have S-ECC (p=0.048). Children whose fathers did not work for pay were significantly more likely to have S-ECC (p=0.05).

Participation in an Aboriginal Head Start Program was not associated with S-ECC; 25.3% attending this early childhood programming had S-ECC compared to 23.8% among those who did not (p=0.78). However, children already attending school were significantly more likely to have S-ECC (33.1% vs. 20.7%, p=0.006; OR=1.9).

### Health status and S-ECC

Overall, there was no association between caregiver ratings of their child's general health and S-ECC; 24.6% of the children rated to be in excellent or very good health had S-ECC versus 27.5% of those who were rated to only be in good or fair health (p=0.69). S-ECC was not associated with other health conditions experienced by the child (Table II). Children who had S-ECC did not experience more ear infections in the previous 12 months than those without S-ECC (1.1±1.1 vs. 1.3±1.3, p=0.39). Despite these findings, children with asthma were at 1.77 times the odds of having S-ECC while those with chronic ear infections were at 1.86 times the odds. The OR for a child with S-ECC having speech or language difficulties was 1.83.

**Table II:** Relationship between S-ECC and childhood health status and conditions

Variable	Number with S-ECC (%)	Number without S-ECC (%)	p	Unadjusted odds ratio (OR)
Parent/caregiver assessment of child's health			0.69	0.86
Excellent–very good	91 (24.6)	279 (75.4)		
Good–fair	11 (27.5)	29 (72.5)		
Asthma			0.16	1.77
Yes	10 (35.7)	18 (64.3)		
No	90 (23.9)	287 (76.1)		
Speech or language difficulties			0.25	1.83
Yes	6 (37.5)	10 (62.5)		
No	93 (24.7)	283 (75.3)		
Chronic ear infections or ear problems			0.20	1.86
Yes	7 (36.8)	12 (63.2)		
No	92 (23.8)	294 (76.2)		

Chi-square analysis.

There was no difference in the prevalence of S-ECC between children who lived in a smoke-free home and those who did not (23.7% vs. 27.4%, p=0.51). However, children of mothers who were reported to have smoked during their pregnancy were significantly more likely to have S-ECC (28.1% vs. 18.6%, p=0.030). In fact, children whose mothers smoked during pregnancy were 1.7 times more likely to have S-ECC.

### Infant feeding practices and S-ECC

A history of having been breastfed appeared to be protective; the prevalence among breastfed children, regardless of duration, was 17.4% versus 31.5% among those who had never been breastfed (p=0.001). The unadjusted OR for S-ECC if a child was breastfed was 0.46. Meanwhile, among children who had been breastfed, there was no difference in the actual duration of breastfeeding between those with and without S-ECC (7.4±9.1 months vs. 8.0±8.6, p=0.70).

**Fig. 1:**
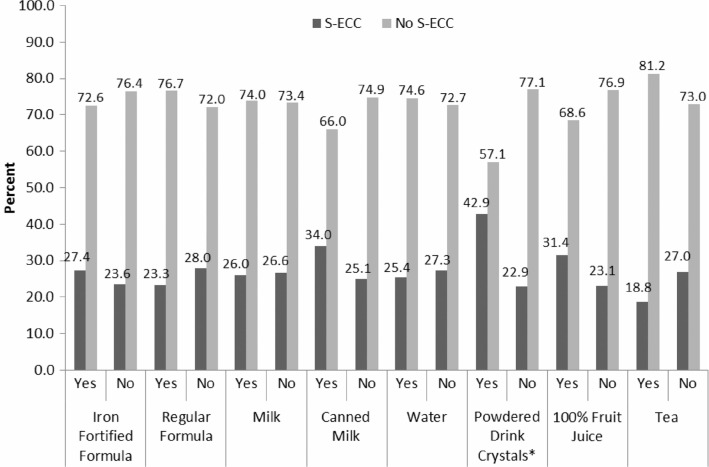
Relationship between bottle contents and S-ECC. Chi-square analysis. *Statistically significant (p≤0.05).

Children who were bottlefed and children who received 100% fruit juice in their bottles tended to be more likely to have S-ECC, although neither finding was significant. Figure 1 reports the association between different bottle contents and S-ECC. The only significant association was that children who had powdered drink crystal beverages in their bottles were significantly more likely to have S-ECC (OR=2.5, p=0.001).

### Dietary habits and S-ECC

Caregivers were also asked about their child's eating habits (Table III). While children consuming milk on a daily basis had lower odds of S-ECC, this was not significant. Specific foods and beverages that were associated with S-ECC included daily juice consumption, soft drinks, sweets and fast food (Table III). Caregivers were also asked about their child's intake of vitamins but no association was found. The prevalence of S-ECC among those who took vitamins was 24.6% versus 25.0% among those who did not (p=0.94).

**Table III:** Relationship between S-ECC and childhood dietary intakes

Variable	Number with S-ECC (%)	Number without S-ECC (%)	p	Unadjusted odds ratio (OR)
Child eats nutritious balanced meals			0.15	0.83
Daily	46 (21.7)	166 (78.3)		
<Daily	46 (25.1)	137 (74.9)		
Fruit			0.47	1.20
Daily	72 (26.2)	203 (73.8)		
<Daily	29 (22.8)	98 (77.2)		
Bread, pasta, rice, grains			0.73	1.09
Daily	76 (25.8)	219 (74.2)		
<Daily	26 (24.1)	82 (75.9)		
Fast food			**0.036**	2.12
Daily	14 (40.0)	21 (60.0)		
<Daily	88 (23.9)	280 (76.1)		
Sweets			**0.010**	2.12
Daily	23 (38.3)	37 (61.7)		
<Daily	77 (22.7)	262 (77.3)		
Milk and milk products			0.19	0.65
Daily	85 (23.9)	270 (76.1)		
<Daily	16 (32.7)	33 (67.3)		
Water			0.89	1.05
Daily	91 (25.4)	267 (74.6)		
<Daily	11 (24.4)	34 (75.6)		
Juice			**0.001**	3.13
Daily	91 (28.9)	224 (71.1)		
<Daily	10 (11.5)	77 (88.5)		
Soft drinks/pop			**0.001**	2.65
Daily	25 (43.1)	33 (56.9)		
<Daily	76 (22.2)	266 (77.8)		

Daily defined as always/almost always.

<Daily defined as sometimes/rarely/never/hardly ever.

Chi-square analysis.

### Barriers to healthcare access and S-ECC

**Fig. 2:**
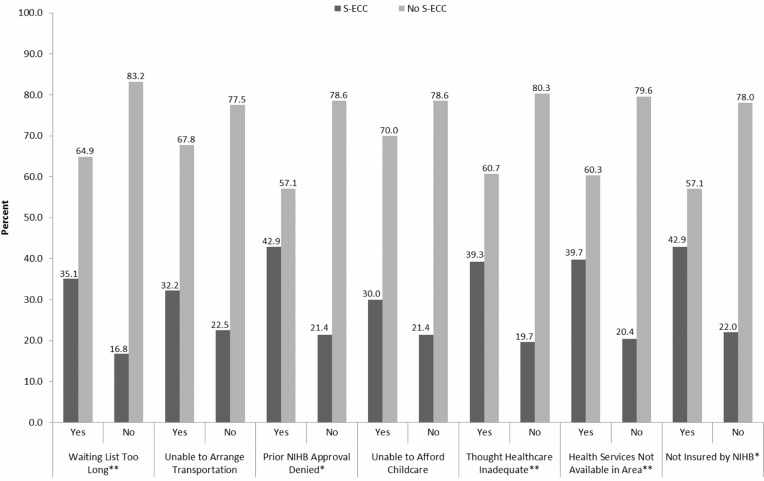
Relationship between barriers to healthcare access and S-ECC. Chi-square analysis. *Statistically significant (p≤0.05). **Statistically significant (p≤0.001).

Caregivers were asked questions about barriers to overall healthcare and access issues for their child (Fig. 2). Those whose caregiver indicated that the waiting list for healthcare services was too long were more likely to have S-ECC (35.1% vs. 16.8%, p<0.001). Likewise, children who reported to have some of their predetermination for services through Health Canada's Non-Insured Health Benefits (NIHB) program previously denied were also more likely to have S-ECC (42.9% vs. 21.4%, p=0.023). The OR for S-ECC among children whose caregiver reported that healthcare services provided were inadequate was 2.63 (39.3% vs. 19.7%, p=0.001). Those indicating that services were unavailable and were not culturally appropriate were also significantly more likely to report that their child had S-ECC (39.7% vs. 20.4% and 46.7% vs. 19.9%, p=0.001 and 0.001, respectively).

### Residential schools and S-ECC

Finally, the relationship between past disruptions to the family unit and S-ECC were explored. No significant relationship was found between S-ECC and whether the child's maternal grandmother or maternal grandfather attended residential school (p=0.30 and 0.50, respectively). Children whose paternal grandmother attended residential school were significantly more likely to have S-ECC (33.7% vs. 19.0%, p=0.009, OR=2.16). Those whose paternal grandfather attended residential school also had a higher prevalence of S-ECC (34.5% vs. 20.8%, p=0.022, OR=2.0).

## Discussion

This study investigated the prevalence of caregiver-reported BBTD/S-ECC and associated risk factors among Manitoba First Nations children. One quarter of preschool children were affected. Among 0–2-year-olds, 18.4% were affected while 30.8% of the 3–5-year-old group had S-ECC. These findings are comparable to national data for preschool children participating in the RHS Phase 2 (26). Results from the RHS Phase 1 survey revealed that fewer infants were affected than among those in the present survey (11.9% vs. 18.7%), suggesting that the onset of severe forms of caries among infants and toddlers may be on the rise (26,27). Caries is a significant issue in Manitoba, with children in 1 northern First Nations community having decay involving nearly 14 out of 20 primary teeth (7). The rate of primary tooth extractions performed in hospital can be as high as 68/1,000 in northern and rural Manitoba (30). Undoubtedly, similar observations can be made in other First Nations communities (11).

The First Nations Oral Health Survey revealed that 85.9% of 3–5-year-olds experienced ECC with an average cumulative decayed, missing, and filled primary tooth (dmft) score of 7.62 (31). Inuit children also suffer from ECC and its rampant subtype, S-ECC (32,33). As many as 85% of Inuit 3–5-year-olds have ECC (dmft=8.2) (33). The burden is also considerable among AIAN children (3,4,34,35). Fortunately, medical and dental professions have recognized this disparity. The American Dental Association has sponsored symposia and the Canadian Pediatric Society and the American Academy of Pediatrics have published a joint position statement on the matter (11,12,36,37). Despite this attention, it is important to avoid stigmatizing S-ECC as a disease of Indigenous populations, as it affects children from different ethnic groups (35,38).

The finding that older children were more likely to have S-ECC is of little surprise. The older a child is the more teeth they have and the longer these teeth have been exposed to cariogenic challenges. While not significant, boys were more likely to have S-ECC. Most studies do not report any significant sex influence on ECC prevalence; however, 1 study involving children attending a community-dental clinic reported that males suffered from ECC more than females (39).

The level of education parents’ possess and their employment status may influence their child's oral health. These indicators reinforce the social determinants of oral health (11). It is well recognized that socioeconomic factors like limited family finances, parental education, and employment opportunities can translate into increased caries experience for young children (11,35,39,40).

One surprising observation was that smoking during pregnancy increased a child's risk for S-ECC. This finding is similar to a large national sample of American preschoolers reporting that maternal prenatal smoking was independently associated with increased caries-risk in offspring (41). Other publications have also reported a link between children's exposure to smoke in the home and an increased risk of caries in their primary teeth (42–44). A possible explanation may be that smokers place less value on healthy living behaviors, like oral hygiene and diet, making them less likely to care for their child's oral health (41,42). Another is that smoking during pregnancy may affect the development of the primary dentition in utero as smoking may result in poorer birth outcomes like prematurity and low birth weight (43). These conditions are risk factors for enamel hypoplasia, which is known to increase the risk for ECC.

Breastfed children were less likely to have S-ECC. Aggregated national data from the RHS Phase 2 also revealed that breastfeeding was associated with a lower prevalence of severe caries (26). Others have also reported that breastfeeding is protective against caries and that there is little evidence suggesting that breastfeeding is a risk factor for ECC and S-ECC (8,41,45). This would imply that many former antecedent terms for ECC that cast breastfeeding in a negative light are incorrect. Efforts to support breastfeeding practices in First Nations communities are welcomed as this may have positive impacts on dental health.

Meanwhile, while not significant in our Manitoba population, bottlefed children were more than twice as likely to develop S-ECC. The association between bottle-feeding and caries is not new and was also reported in the national RHS Phase 2 report (26). Bottle contents are often to blame as they contain fermentable carbohydrates that are cariogenic. Constant sipping means that bottle contents are in constant contact with teeth. Those who choose to bottle-feed must be aware that bottles should only be reserved for feeding time. If a child must go to bed with a bottle, it should only contain pure water. In our Manitoba RHS cohort, the only beverage put in bottles that was found to be significantly associated with severe caries was drink crystals, such as Kool-Aid. However, the national data revealed that putting juice, tea, iron fortified formula, canned milk, soft drinks, and drink crystals in the bottle significantly increased the risk (26). While some inappropriate infant-feeding practices definitely contribute to the problem, we identified many more risk factors in our study population. Therefore, while the term BBTD is easily recognized, it should be abandoned as it only highlights the harm arising from inappropriate bottle feeding and contents rather than shedding light on numerous other health and social determinants that influence primary teeth.

Children consuming soft drinks, sweets, juice, and fast food daily were more likely to have S-ECC. These snacks are convenient and readily available in communities. It is well recognized that the more frequent the consumption of these and other cariogenic foods and beverages, the higher the risk of decay. Snack foods, such as chips, cookies, candy, and drinks like pop and juice, have all been linked with caries (46). The reality is that the cost of nutritious food, including milk, is often prohibitive in northern regions of Canada (7). Attempts should be made to make nutritious foods available and affordable (11).

Even barriers and access to overall healthcare can impact oral health. Caregivers who reported that the wait for healthcare services was too long or not available in their home community were more likely to have a child with S-ECC. Early access to preventive oral health care is important yet we are unaware of the number of dentists working in First Nations communities. A potential lack of access greatly impacts parents’ ability to obtain anticipatory guidance and preventive dental care for their child. The Canadian Dental Association recommends a first dental visit within 6 months of the eruption of the first tooth and no later than 12 months of age (47). Delayed first visits are associated with increased risk of dental problems (39). It is unknown how many dental practitioners working in First Nations communities follow this recommendation, despite the fact that most First Nations children living on-reserve have NIHB dental benefits. However, we need to dispel the myth that dental insurance translates into access to care (48). Many other barriers to oral health care exist and need to be addressed to improve timely and regular access to both prevention and treatment. Many First Nations children are still unable to obtain a dental home (4). Caregivers’ identified concerns over barriers to care and access were associated with their child's caries experience. Recent recommendations call for cultural proficiency training for health providers and increasing the capacity of Aboriginal oral health professionals (11,12).

Children with a family history of residential school experience had a higher prevalence of S-ECC. While we cannot offer a direct explanation, it is well recognized that colonization and attempts by the Canadian government to force assimilation of First Nations children through residential schools has had long-lasting negative effects on families and communities (49). The traumatic separation of young children from their homes and family units led to the destruction of the family and loss of traditions and culture (49). The resultant disruptions and loss of traditional knowledge has affected the overall health and well-being of those who endured residential schools, their children and grandchildren (49). Theoretically, this has likely also contributed to the existing oral health disparity seen in First Nations persons. Apart from the traditional health determinants, there are particular determinants unique to Indigenous people that also influence their well-being, including loss of culture, self-determination, inequitable access to healthcare, and social environments and social supports (49).

Efforts to improve the oral health of First Nations children need to be informative, non-judgmental and culturally appropriate. Community-based initiatives that rely on community development principles are appropriate (11,12,50). While some clinical trials involving Indigenous children have used chemotherapeutic agents to prevent or arrest caries, they alone do not appear to be the silver bullet (2,51). Rather, multi-pronged approaches that also address determinants of health may offer hope (4). Potential strategies to consider include motivational interviewing, improving access to early visits, access to fluoridated water and nutritious food choices, along with antimicrobials and applying remineralizing agents to existing caries lesions (4,11,36,47,52). Regardless, we need to find an appropriate metric, like monitoring the volume of children requiring dental surgery, to measure the success of such interventions for these children (2).

This study has some limitations. Data were derived from interviews with caregivers. While this study did not include clinical examinations, the findings are important as self-reported oral health has been be correlated with clinical status (53). Our group has previously reported that assessments of children's teeth are associated with clinical findings (40). Further, participants were only asked about whether their child was affected by BBTD, which is synonymous with S-ECC. However, the RHS survey did not ask about overall caries experience (i.e. ECC). Therefore, we are unable to determine the caregiver-reported prevalence of ECC, but rather more severe forms. This prohibits direct comparisons with other published studies of ECC in Canadian children. Further, as this was based on self-reports, the true burden of caries may be underestimated. Many of the questions posed to caregivers were also retrospective in nature and subject to recall bias. We were also unable to assess associations between S-ECC and other variables because of small cell sizes.

## Conclusions

One quarter of First Nations children in Manitoba were affected with S-ECC. Risk factors for caries included the child's age, caregiver education and employment levels, and maternal smoking during pregnancy. Breastfeeding was found to be protective against severe caries, while dietary intakes of sweets, fast foods and sugary drinks increased the risk. Barriers and access to care and disruptions to family and culture also appeared to be associated with an increased prevalence of S-ECC. This local evidence can and should be used to help inform future caries prevention and risk minimization activities in these Manitoba communities.
